# Neurofilament light protein in blood as a potential biomarker of neurodegeneration in Huntington's disease: a retrospective cohort analysis

**DOI:** 10.1016/S1474-4422(17)30124-2

**Published:** 2017-08

**Authors:** Lauren M Byrne, Filipe B Rodrigues, Kaj Blennow, Alexandra Durr, Blair R Leavitt, Raymund A C Roos, Rachael I Scahill, Sarah J Tabrizi, Henrik Zetterberg, Douglas Langbehn, Edward J Wild

**Affiliations:** a UCL Institute of Neurology, London, UK; b Department of Psychiatry and Neurochemistry, Institute of Neuroscience and Physiology, Sahlgrenska Academy at the University of Gothenburg, Mölndal, Sweden; c Clinical Neurochemistry Laboratory, Sahlgrenska University Hospital, Mölndal, Sweden; d Centre for Molecular Medicine and Therapeutics, University of British Columbia, Vancouver, BC, Canada; e Department of Neurology, Leiden University, Leiden, Netherlands; f Institut du Cerveau et de la Moelle épinière (ICM), Sorbonne Universités, UPMC University Paris 06, UMRS_1127, INSERM, U 1127, CNRS, UMR 7225, APHP, Genetics Department, Pitié-Salpêtrière University Hospital, Paris, France; g Department of Psychiatry, Carver College of Medicine, University of Iowa, Iowa City, IA, USA

## Abstract

**Background:**

Blood biomarkers of neuronal damage could facilitate clinical management of and therapeutic development for Huntington's disease. We investigated whether neurofilament light protein NfL (also known as NF-L) in blood is a potential prognostic marker of neurodegeneration in patients with Huntington's disease.

**Methods:**

We did a retrospective analysis of healthy controls and carriers of CAG expansion mutations in *HTT* participating in the 3-year international TRACK-HD study. We studied associations between NfL concentrations in plasma and clinical and MRI neuroimaging findings, namely cognitive function, motor function, and brain volume (global and regional). We used random effects models to analyse cross-sectional associations at each study visit and to assess changes from baseline, with and without adjustment for age and CAG repeat count. In an independent London-based cohort of 37 participants (23 *HTT* mutation carriers and 14 controls), we further assessed whether concentrations of NfL in plasma correlated with those in CSF.

**Findings:**

Baseline and follow-up plasma samples were available from 97 controls and 201 individuals carrying *HTT* mutations. Mean concentrations of NfL in plasma at baseline were significantly higher in *HTT* mutation carriers than in controls (3·63 [SD 0·54] log pg/mL *vs* 2·68 [0·52] log pg/mL, p<0·0001) and the difference increased from one disease stage to the next. At any given timepoint, NfL concentrations in plasma correlated with clinical and MRI findings. In longitudinal analyses, baseline NfL concentration in plasma also correlated significantly with subsequent decline in cognition (symbol-digit modality test *r*=–0·374, p<0·0001; Stroop word reading *r*=–0·248, p=0·0033), total functional capacity (*r*=–0·289, p=0·0264), and brain atrophy (caudate *r*=0·178, p=0·0087; whole-brain *r*=0·602, p<0·0001; grey matter *r*=0·518, p<0·0001; white matter *r*=0·588, p<0·0001; and ventricular expansion *r*=–0·589, p<0·0001). All changes except Stroop word reading and total functional capacity remained significant after adjustment for age and CAG repeat count. In 104 individuals with premanifest Huntington's disease, NfL concentration in plasma at baseline was associated with subsequent clinical onset during the 3-year follow-up period (hazard ratio 3·29 per log pg/mL, 95% CI 1·48–7·34, p=0·0036). Concentrations of NfL in CSF and plasma were correlated in mutation carriers (*r*=0·868, p<0·0001).

**Interpretation:**

NfL in plasma shows promise as a potential prognostic blood biomarker of disease onset and progression in Huntington's disease.

**Funding:**

Medical Research Council, GlaxoSmithKline, CHDI Foundation, Swedish Research Council, European Research Council, Wallenberg Foundation, and Wolfson Foundation.

## Introduction

Huntington's disease is a slowly progressive neurodegenerative disorder for which no proven disease-modifying treatments yet exist. Knowledge of its genetic cause, CAG repeat expansions in the *HTT* gene leading to the formation of mutant huntingtin (mHTT), has enabled focused study of the disease and the development of advanced therapeutics targeting known aspects of its pathobiology.^1^

Although extensive efforts have established well characterised clinical, cognitive, and neuroimaging biomarkers of progression,^2^, ^3^, ^4^, ^5^ few biochemical markers have been identified that enable direct assessment of relevant aspects of pathology.^6^, ^7^ No prognostic biomarkers for assessing neuronal damage, disease progression, or therapeutic response have been validated, which limits the ability to test novel therapeutics, especially in mutation carriers with premanifest Huntington's disease for whom treatment is most likely to result in long-term meaningful benefits. Accessible, reliable, biochemical markers would greatly facilitate the development of novel therapeutics for Huntington's disease.^1^

Many potential markers in CSF have been proposed, but only a few (eg, mHTT itself, microtubule-associated protein tau, and chitinase-3-like protein 1) have shown associations with clinical phenotype beyond established predictors, such as age and *HTT* CAG triplet repeat count.^7^, ^8^, ^9^, ^10^ None of the potential biomarkers has been studied longitudinally.^7^ Moreover, CSF is more difficult and expensive to obtain than other fluids, but no substance detectable in a highly accessible biofluid, such as blood, has robustly reflected Huntington's disease-related alterations due to CNS pathology, either in cross-sectional or longitudinal studies. We previously showed that mHTT concentrations in blood leucocytes were associated with clinical severity cross-sectionally,^11^ but that this was probably due to peripheral mHTT production rather than CNS pathology. This and other such markers of peripheral pathology,^12^ therefore, are poor candidates for trials involving direct CNS delivery of disease-modifying agents.

**Research in context**

**Evidence before this study**

We searched PubMed by use of the MeSH terms “([intermediate filaments] AND [nerve degeneration OR Huntington disease OR Alzheimer disease OR Parkinson disease OR Pick disease of the brain OR frontotemporal dementia OR amyotrophic lateral sclerosis OR supranuclear palsy, progressive] AND [blood OR plasma OR serum OR cerebrospinal fluid] AND [humans])” and their natural language variants for human studies of neurofilament light protein (NfL, also known as NF-L) in neurodegenerative disorders. Four small cross-sectional studies reported raised concentrations of NfL in the CSF of people with Huntington's disease, and four longitudinal studies had assessed blood NfL concentrations in other disorders. The largest longitudinal study of people with neurodegenerative disease measured NfL in 174 patients with progressive supranuclear palsy over 1 year. The largest study of neurodegenerative disease onset was done in 34 *GRN* mutation carriers with premanifest frontotemporal dementia, among whom only two had disease progression during follow-up. The association between concentrations of NfL in plasma has never been reported in people with Huntington's disease. We investigated whether NfL concentrations in plasma could serve as a potential prognostic marker of neurodegeneration in a genetically homogeneous cohort of *HTT* CAG expansion mutation carriers, including a substantial subgroup with premanifest disease, followed up over 3 years.

**Added value of this study**

Increased concentrations of NfL in plasma were seen throughout the course of Huntington's disease and, after controlling for age and CAG repeat count, were independently associated with cognitive and motor dysfunction and global and regional brain volume at any given timepoint. We also found a strong association between increased NfL concentration and CAG repeat count, which suggests a firm link between this factor and the genetic basis of Huntington's disease. In *HTT* mutation carriers with premanifest disease at baseline, increased concentration of NfL in plasma at baseline was associated with subsequent clinical onset beyond the known prognostic variables of age, CAG repeat count, and brain volume; such an association has not previously been found for other potential biofluid biomarkers. Associations were also seen with disease progression assessed by cognitive, functional, and brain atrophy measures. In an independent cohort of 37 individuals (23 *HTT* mutation carriers and 14 controls), we showed a strong correlation between raised NfL concentrations in plasma and matched CSF.

**Implications of all the available evidence**

Measurement of NfL in plasma could be useful to assess the risk of Huntington's disease onset and progression beyond currently known prognostic factors. Our findings in individuals with premanifest Huntington's disease suggest that NfL has potential as a biomarker in the preclinical phases of other neurodegenerative diseases. The availability of accessible, reliable biochemical markers might also be useful in assessing novel therapeutics. Finally, this study affirms the benefits of systematically studying genetically homogeneous cohorts to assess the earliest changes in neurodegeneration.

Neurofilament light protein (NfL, also known as NF-L) is the smallest of three subunits that make up neurofilaments, which are major components of the neuronal cytoskeleton. NfL is released from damaged neurons. Concentrations in CSF are increased in people with neurodegenerative diseases, including Alzheimer's disease,^13^ amyotrophic lateral sclerosis,^13^ and frontotemporal dementia.^13^, ^14^ Four small-scale cross-sectional studies found raised concentrations of NfL in the CSF of individuals with Huntington's disease,^8^, ^15^, ^16^, ^17^ and we have shown a close association between increased concentrations of NfL and mHTT in CSF.^8^

NfL is detectable in blood plasma or serum. Cross-sectional studies have shown increased concentrations in blood in people with frontotemporal dementia,^18^ Alzheimer's disease, amyotrophic lateral sclerosis,^19^ and atypical parkinsonism,^20^ and in longitudinal studies of those with frontotemporal dementia,^21^, ^22^ amyotrophic lateral sclerosis,^23^ and progressive supranuclear palsy.^24^ A 1-year study followed up 147 patients with progressive supranuclear palsy, but these cohorts were genetically and pathologically heterogeneous and only a few included premanifest individuals, in small numbers.^24^ NfL concentrations in blood have not been reported in people with Huntington's disease.

We aimed to investigate whether NfL in plasma could act as a potential prognostic marker of neurodegeneration and disease progression for Huntington's disease. The study involved the 366 participants of the TRACK-HD cohort,^5^, ^25^ who had been assessed by standardised blood sampling, clinical testing, and MRI annually over 3 years. This cohort offers a unique resource for studying neurodegeneration in a genetically uniform disease cohort and for assessing changes at different stages of disease, including in the premanifest phase. We tested the hypotheses that NfL concentrations would be raised in individuals with Huntington's disease, would increase as disease progressed, and that concentrations would correlate with disease onset in *HTT* mutation carriers with premanifest Huntington's disease and with clinical progression in those with manifest disease, therefore acting as an indicator of neurodegeneration. We additionally investigated whether concentrations of NfL in CSF would correlate with those in plasma.

## Methods

### Study design and participants

We did a retrospective study involving 366 participants enrolled in the TRACK-HD study at four international study sites in 2008. Participants were assessed annually with standardised 3-Tesla T1 volumetric MRI, clinical, cognitive, quantitative motor, and neuropsychiatric assessments, as previously described.^25^ The study protocol is available online. At enrolment, participants with *HTT* CAG expansion mutations were classified as having premanifest or manifest Huntington's disease based on the Unified Huntington's Disease Rating Scale (UHDRS) Total Motor Score (TMS). Participants with premanifest disease were further separated into two subgroups of early and late premanifest disease (termed preHD A and preHD B in some other TRACK-HD publications), with the group median for predicted number of years to onset of manifest disease (10·8) as the threshold. Using the UHDRS Total Functional Capacity (TFC) score, patients with manifest Huntington's disease were separated into subgroups with clinical stage 1 (TFC score >10) and stage 2 (TFC score 7–10) disease. Controls were healthy partners or siblings of *HTT* mutation carriers. All human studies are compliant with the Declaration of Helsinki and approved by local ethics committees, and all participants gave written informed consent.

### Clinical and imaging assessments

T1 volumetric MRI scans were subjected to rigorous quality control followed by analysis by operators unaware of participants' statuses at specialist image analysis sites with use of optimised, standardised techniques, as previously described.^5^ Briefly, cross-sectional putamen volumes were calculated by automated segmentation; whole-brain, caudate, lateral ventricles, and total intracranial volumes by semiautomated segmentation; and grey-matter and white-matter volumes by voxel-based morphometry. Longitudinal changes in whole-brain, ventricles, and caudate were calculated with the boundary shift integral technique, and changes in grey-matter and white-matter volume were assessed with voxel compression mapping within voxel-based morphometry segmentations.^5^ All cross-sectional imaging measures were calculated as a percentage of total intracranial volume. Cognitive function was assessed with the Symbol-Digit Modality Test (SDMT) and Stroop Word Reading (SWR) task, and clinical severity was assessed with the UHDRS TMS and TFC.^25^

### NfL quantification in plasma

At each TRACK-HD visit, blood was collected in BD Vacutainer tubes containing edetic acid (Franklin Lakes, NJ, USA.). Samples were processed onsite to isolate plasma, as previously described.^26^ Plasma samples were frozen, stored at −80°C, then shipped frozen for analysis by operators unaware of participants' disease statuses. NfL concentrations were quantified with an ultrasensitive single-molecule array method.^18^ All NfL values were within the linear ranges of the assays.

### CSF cohort

A London-based independent cohort of 37 participants (14 controls, three *HTT* mutation carriers with premanifest disease [UHDRS diagnostic confidence scores <4], and 20 participants with manifest Huntington's disease) underwent CSF and plasma collection standardised for diet, time of day, clinical procedures, and processing.^8^ Blood was collected within 30 min of CSF in sodium heparin cell-preparation BD Vacutainer tubes and processed to isolate plasma. NfL concentrations in CSF were quantified with a commercial ELISA, used according to the manufacturer's protocol (UmanDiagnostics, Umeå, Sweden).^8^

### Statistical analysis

Analysis of plasma NfL in TRACK-HD was done per a prespecified statistical plan that was designed with and done by a statistician experienced in analysing the TRACK-HD dataset (DL). Outcomes of interest (UHDRS TMS, UHDRS TFC, SWR, SDMT, and volumes of whole-brain, caudate, putamen, lateral ventricles, grey matter, and white matter) were chosen because they had the strongest effect sizes in TRACK-HD.^5^ Clinical and imaging data were paired to NfL concentrations in plasma at each study visit. Longitudinal changes were calculated over the longest available period and converted to annualised rates.

NfL concentrations in plasma were non-normally distributed because of biologically plausible higher values. Natural log-transformation produced plausibly normal distributions and was used for all analyses. Longitudinal changes in UHDRS TMS were calculated by square-root transformation of cross-sectional values.

ANOVA was used to compare groups at baseline. Other cross-sectional associations were assessed for individuals' baseline and follow-up measurements in linear models that included a random participant effect. Sex and study site were not significantly associated with NfL and, therefore, were not included as covariates. When controlling for the interacting effects of age and CAG repeat count (known prognostic factors for progression of Huntington's disease), we used a polynomial model that included linear and squared terms for variables and interactions between them, since in our observations of other Huntington's disease phenomena such higher order terms have often been significantly associated with progression.

Longitudinal changes in NfL concentrations were assessed with correlated random intercept and slope models. UHDRS TFC changes are negligible in individuals with premanifest Huntington's disease and, therefore, longitudinal analyses of changes in this measure were restricted to individuals with manifest Huntington's disease. For simplicity, we describe longitudinal associations with Pearson's correlations between cross-sectional NfL concentrations and other outcomes, and between baseline NfL concentrations and the annualised rates of change in other outcomes, calculated from data available at the last available assessment. However, all p values are derived from analogous random effects repeated measurement models, which allow proper inference but lack unambiguously defined corresponding correlation statistics.

We used Cox proportional hazard survival modelling to calculate hazard ratios (HRs) and 95% CIs for the correlation between baseline NfL concentration and subsequent onset of Huntington's disease within 3 years in premanifest HTT mutation carriers. The small number of confirmed new diagnoses (n=18) precluded simultaneous inclusion of multiple covariates, but their pattern was consistent with the proportional odds assumption. Thus, other known risk factors previously identified in the TRACK-HD data^5^, ^27^ were controlled separately to assess non-redundancy of NfL concentrations as a risk factor.

The data for the independent CSF cohort were analysed without log transformation. Participants with premanifest and manifest Huntington's disease were pooled to create one Huntington's disease group. We used Wilcoxon's rank sum test to compare groups, and Pearson's correlation coefficient and partial correlations with bootstrap estimates of SEs with 1000 replications to assess associations between variables.

The threshold for statistical significance for all analyses was p<0·05. Where prominent outliers remained after normalisation, they were excluded and analyses were repeated to evaluate their influence. The analysis was carried out in SAS (v9.4, SAS Institute Inc, Cary, NC, USA) using SAS/STAT 14.1.

### Role of the funding source

The funders of the study had no role in the design, data collection, data analysis, data interpretation, or writing of the report. The corresponding author had full access to all the data in the study and had final responsibility for the decision to submit for publication.

## Results

Of 366 TRACK-HD participants, baseline and follow-up plasma samples were available from 298 (81%) who completed the 3-year TRACK-HD study (97 controls and 201 *HTT* mutation carriers—58 with early premanifest and 46 with late premanifest disease, and 66 with stage 1 and 31 with stage 2 Huntington's disease). 293 had paired plasma samples from the 3-year follow-up visit, four from the 2-year visit, and one from the 1-year visit. The demographic and clinical characteristics of participants at baseline are presented in the appendix.

**Figure 1:**
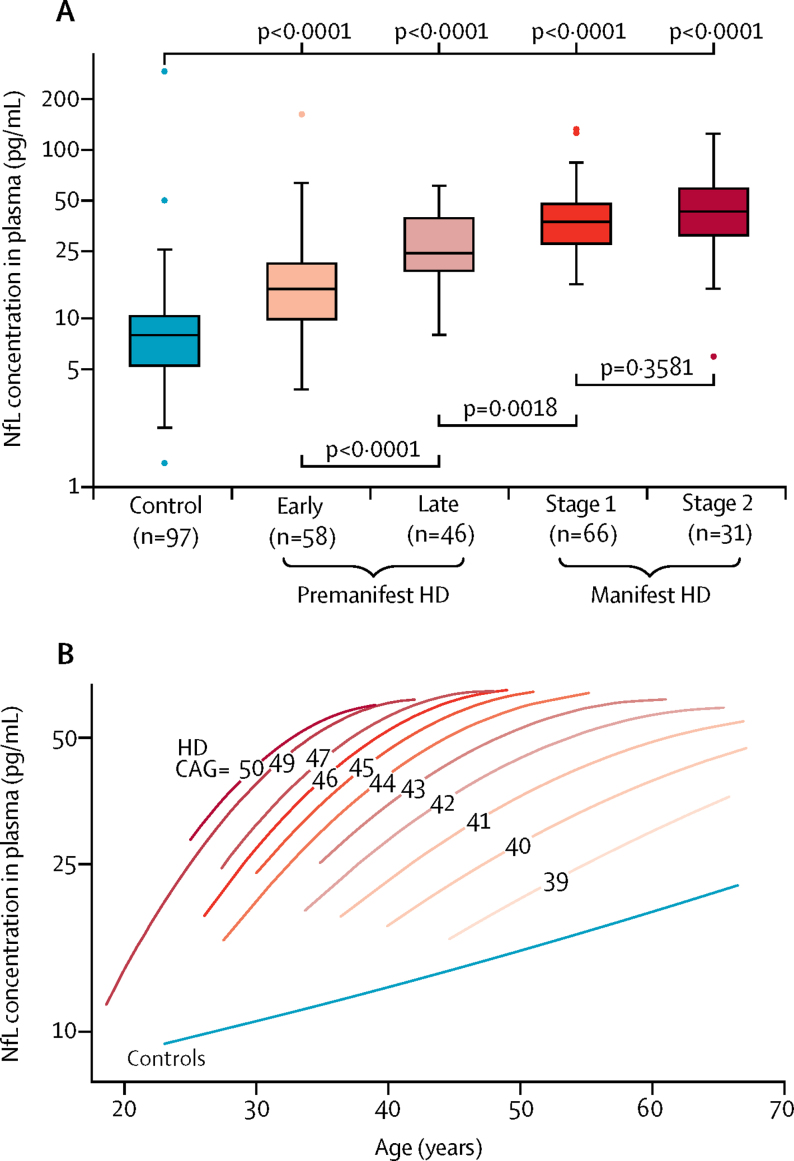
Associations between NfL concentrations in plasma and disease stage, age, and CAG triplet repeat count (A) Baseline NfL concentrations in plasma, by disease stage. Boxes show first and third quartiles, the central band shows the median, and the whiskers show data within 1·5 IQR of the median. The dots represent outliers. Data were log transformed for comparisons. (B) Associations between NfL concentration in plasma, age, and CAG repeat count, modelled with a polynomial function of age, CAG repeat counts, their squares, and their interactions in 201 *HTT* mutation carriers and 97 controls. The lines show quadratic fit for all participants with a given CAG repeat count or all controls. Each increase in CAG repeat count was associated with higher and more steeply rising NfL concentrations in plasma. Predicted values are truncated at the vertical inflection point of the parabola. Datapoints for each individual CAG repeat count and for controls are provided in the appendix. HD=Huntington's disease mutation carriers. CAG=mutation carriers' CAG repeat counts.

Concentrations of NfL in plasma at baseline were 2·6 times higher before log transformation in *HTT* mutation carriers than in controls (mean 3·63 [SD 0·54] log pg/mL *vs* 2·68 [0·52] log pg/mL, p<0·00001). Baseline NfL concentrations in plasma were significantly higher in all disease stage subgroups among *HTT* mutation carriers than those in controls (all p<0·0001, figure 1 and appendix). Additionally, NfL concentrations differed significantly with increasing disease stage except stage 2 versus stage 1 Huntington's disease (figure 1 and appendix). The stage 2 manifest subgroup did, however, differ significantly from the early premanifest subgroup (mean difference 0·785 [SE 0·113], p<0·0001) and the late premanifest subgroup (0·348 [0·102], p=0·0017).

We found positive associations between NfL concentrations in plasma and age in controls and all Huntington's disease subgroups. In controls, the association was roughly linear (slope 0·02 log pg/mL per year [SE 0·0042], p<0·0001; figure 1B). In *HTT* mutation carriers there was a significant positive association between NfL concentration in plasma and the CAG-age product, which measures the extent of exposure to the effects of the CAG expansion (appendix),^4^ but the non-linear association with age and CAG triplet repeat count was best described by CAG-repeat-count-dependent quadratic functions of age (appendix). The association is clear when each CAG count is considered separately (figure 1B, appendix) and shows that for a given age, overall NfL concentrations in plasma increased with increasing CAG repeat count, and that the steepness of the slopes declined with increasing age. Thus, maximum predicted NfL concentrations at older ages became similar.

**Figure 2:**
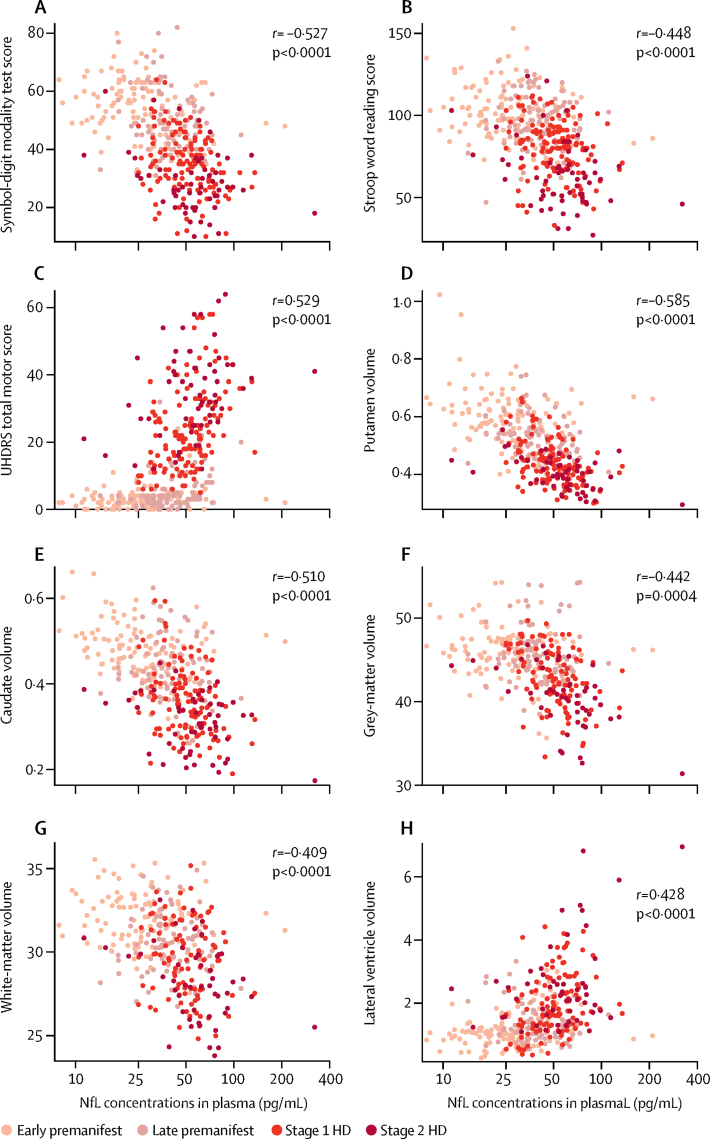
Associations between NfL concentrations in plasma at baseline and cross-sectional measures of cognitive function, motor impairment, and brain volume on MRI (A, B) Association with cognitive scores. (C) Association with motor function. (D–H) Associations with global and regional brain volumes, expressed as percentages of total intracranial volume. UHDRS=Unified Huntington's Disease Rating Scale.

In *HTT* mutation carriers, NfL concentrations in plasma at baseline were negatively associated with cognitive score on the SDMT and SWR and with the MRI measures of brain volume for putamen, caudate, grey matter, and white matter (higher NfL values were associated with smaller brain volumes, figure 2). These associations remained significant after adjustment for the combined effects of age and CAG repeat count (r=–0·293, p<0·0001 for SDMT, r=–0·239, p=0·0042 for SWR; r=–0·286, p<0·0001 for putamen, r=–0·187, p=0·017 for caudate, r=–0·198, p=0·0004 for grey matter, and r=–0·121, p=0·048 for white matter; appendix). The exception was whole-brain volume, which showed a significant negative association before adjustment (*r*=–0·447, p<0·0001), but not after (*r*=–0·120, p=0·150). Significant positive associations were seen with UHDRS TMS (higher NfL values were associated with worse motor performance) and lateral ventricle volume (higher NfL values were associated with larger ventricles; figure 2). These associations persisted after adjustment for age and CAG count (r=0·246, p<0·0001 for TMS, r=0·260, p<0·0001 for ventricular volume; appendix).

Concentrations of NfL in plasma increased significantly from baseline in individuals with premanifest Huntington's disease, by 0·060 log pg/mL per year (SE 0·012, p<0·0001), and in those with manifest Huntington's disease, by 0·026 log pg/mL per year (0·0129, p=0·0442). No change was seen in controls (0·018 log pg/mL per year [0·0128], p=0·171). The rate of increase was significantly greater in *HTT* mutation carriers with premanifest Huntington's disease than in controls (0·043 log pg/mL per year [0·018], p=0·0161) but did not differ significantly between those with premanifest and manifest Huntington's disease (0·034 log pg/mL per year [0·018], p=0·0547) or between those with manifest Huntington's disease and controls (0·009 log pg/mL per year [0·018], p=0·630). The greater rate of increase in premanifest Huntington's disease is consistent with the non-linear associations between NfL concentrations, age, and CAG repeat count.

**Figure 3:**
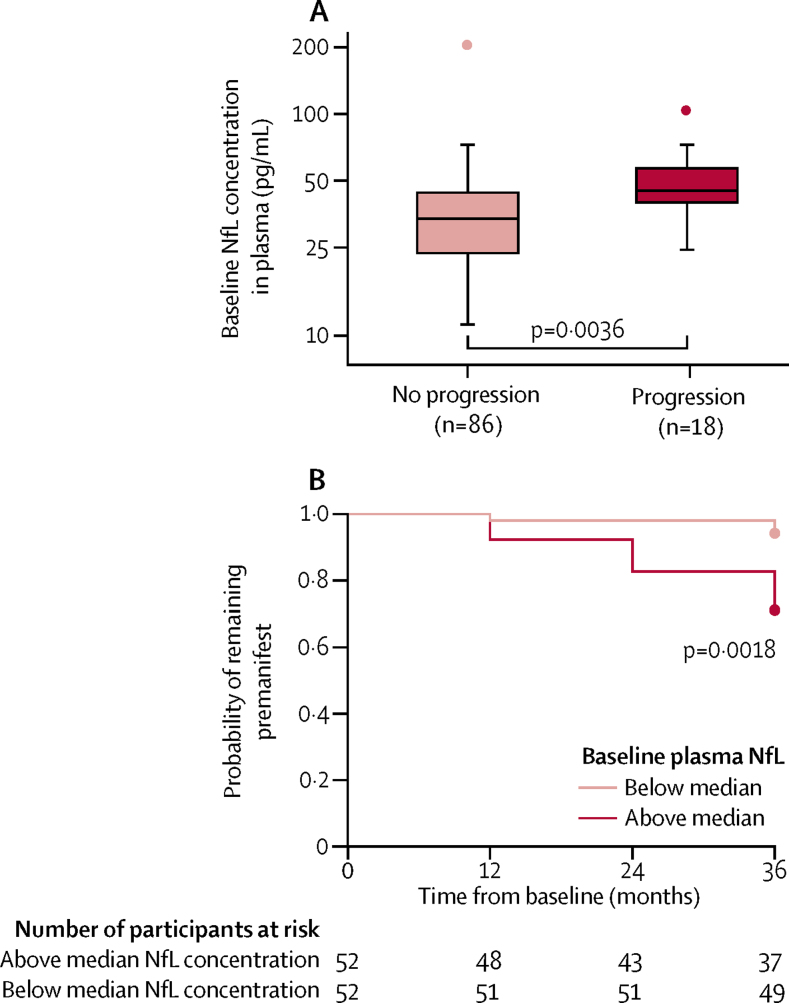
Association between baseline NfL concentration in plasma and progression to manifest Huntington's disease in *HTT* mutation carriers who were premanifest at baseline (A) NfL concentration in plasma at baseline by disease progression status at 3 years. Boxes show first and third quartiles, the central band shows the median, and the whiskers show data within 1·5 IQR of the median. The dots represent outliers. (B) Kaplan-Meier plot showing longitudinal survival in the premanifest state among *HTT* mutation carriers with NfL concentrations in plasma greater or less than the median. The Cox proportional hazards model is the more sensitive of the two models presented here.

18 (17%) of 104 *HTT* mutation carriers with premanifest disease at baseline were newly diagnosed as having manifest Huntington's disease during the TRACK-HD study. The association between baseline NfL concentration in plasma and subsequent disease onset was significant (HR 3·29 per 1·0 log pg/mL, 95% CI 1·48–7·34, p=0·0036; figure 3), and remained so after adjustment for age, CAG repeat count, and their interactions (3·03 per 1·0 log pg/mL, 1·07–8·60, p=0·0371) and for each baseline brain volume measure (appendix).^27^ A receiver operating characteristic curve for risk of diagnosis within 3 years showed that mean sensitivity and specificity were highest when NfL concentration in plasma was 3·61 log pg/mL at baseline (appendix), which is close to the median value among participants with premanifest Huntington's disease at baseline of 3·69 log pg/mL.

**Figure 4:**
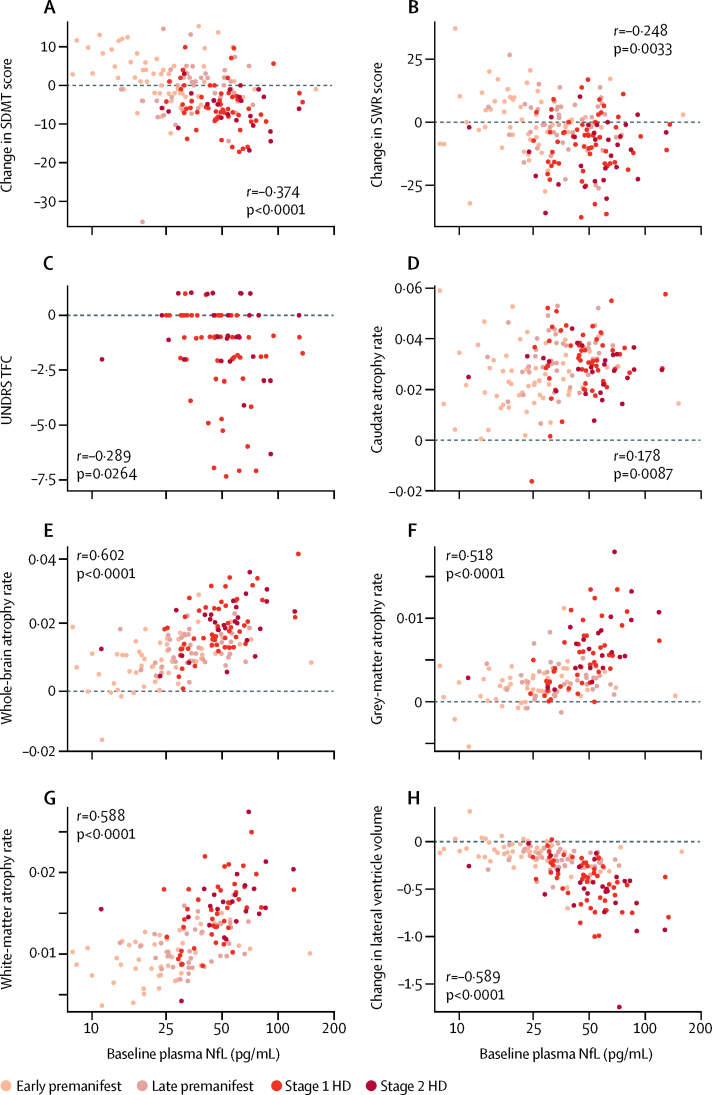
Associations between baseline NfL concentration in plasma and longitudinal change in cognitive, motor, and functional decline and brain atrophy (A, B) Associations with cognitive scores. (C) Association with functional capacity. (D–H) Associations with global and regional brain volumes, expressed as percentages of total intracranial volume. By convention, negative values for change in lateral ventricle volumes indicate ventricular expansion (ie, brain atrophy). SDMT=Symbol-Digit Modality Test Score. SWR=Stroop Word Reading score. UHDRS TFC=Unified Huntington's Disease Rating Scale Total Functional Capacity score. HD=Huntington's disease mutation carriers.

We found significant associations between NfL concentration in plasma at baseline and subsequent decline in cognition and total functional capacity (figure 4). After adjustment for age and CAG repeat count, NfL concentration remained an independent prognostic factor for decline in SDMT (*r*=–0·173, p<0·0001) but not SWR (*r*=–0·040, p=0·4057) or UHDRS TFC (*r*=–0·151, p=0·1107). No association was found with change in motor score (*r*=0·112, p=0·0592). We found positive associations with atrophy of the caudate, whole-brain, grey matter, and white matter and with ventricular expansion (figure 4). These associations remained significant after adjustment for age and CAG repeat count (*r*=0·199, p=0·0043 for caudate, *r*=0·320, p<0·0001 for whole-brain; *r*=0·242, p=0·019 for grey matter; *r*=0·327, p<0·0001 for white matter; and *r*=–0·323, p=0·0002 for ventricular expansion).

Diagnostic status is an obvious prognostic indicator in Huntington's disease and, therefore, we additionally assessed whether NfL concentrations in plasma at baseline were associated with change in brain volumes after controlling for baseline diagnostic status, age, and CAG repeat count. We found independent associations with changes in whole-brain, grey-matter, and white-matter volumes and with ventricular expansion. Furthermore, the associations were independently significant in the premanifest and manifest Huntington's disease subgroups for whole-brain atrophy, grey-matter atrophy, and lateral ventricular expansion (appendix), but for all features where the difference between these subgroups was significant, the prognostic value was a stronger indicator in manifest than in premanifest Huntington's disease.

**Figure 5:**
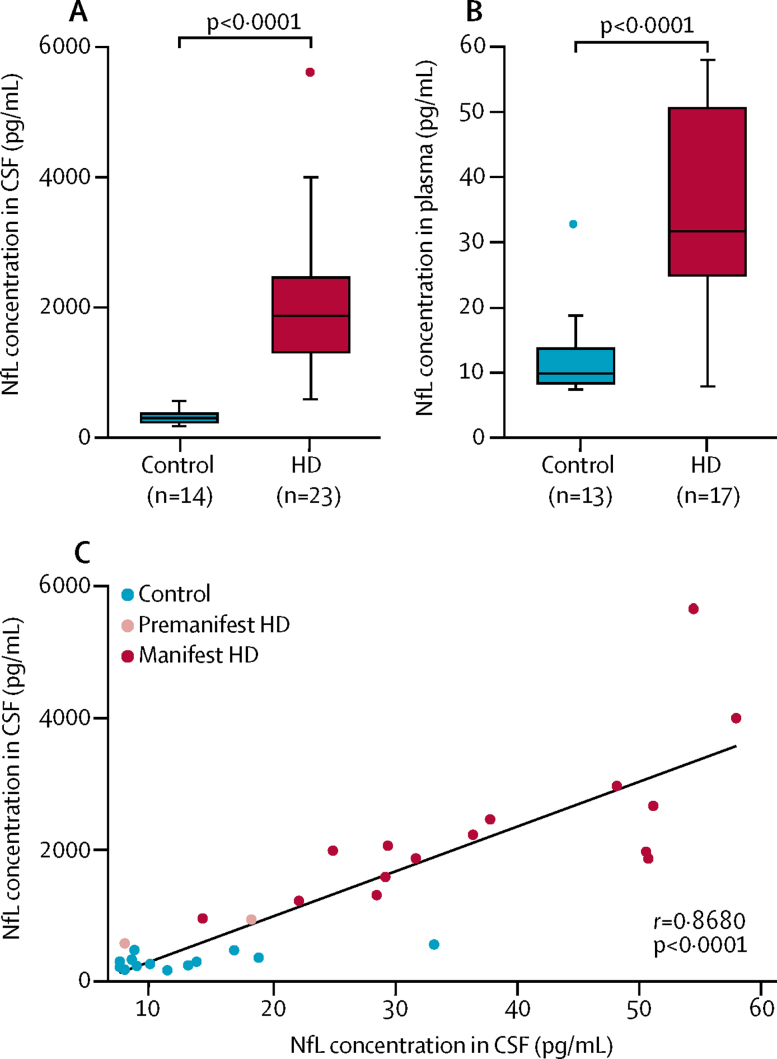
NfL concentrations in paired CSF and plasma samples Concentrations in CSF (A) and plasma (B) in *HTT* mutation carriers and controls. Boxes show first and third quartiles, the central band shows the median, and the whiskers show data within 1·5 IQR of the median. The dots represent outliers. (C) Correlation between NfL concentration in CSF and plasma. HD=Huntington's disease mutation carriers.

The median concentration of NfL in CSF in the 37 participants in the independent CSF cohort was significantly higher in *HTT* mutation carriers than in controls (1871 pg/mL, IQR 1312–2461 *vs* 300 pg/mL, 234–368, figure 5), and in mutation carriers a positive association was seen with UHDRS TMS (*r*=0·4815, p=0·012). In matched plasma samples from 30 participants, the median NfL concentration was also significantly higher in *HTT* mutation carriers than in controls (31·7 pg/mL, IQR 24·9–50·6 *vs* 9·9 pg/mL, 8·4–13·7, figure 5), which in mutation carriers was also positively associated with UHDRS TMS (*r*=0·709, p<0·0001). In keeping with CNS origin, the median NfL concentration in CSF was 46·4 times higher than that in plasma. The ratio differed significantly between controls and *HTT* mutation carriers (30·1 *vs* 62·11, p<0·0001), but there was a positive association between concentrations in plasma and CSF (figure 5).

## Discussion

In this genetically homogeneous cohort of *HTT* mutation carriers, uniquely large and well characterised among neurodegenerative disease cohorts, we found that NfL concentrations in plasma are increased compared with controls. Additionally, concentrations increased with advancing disease—most steeply in participants with premanifest Huntington's disease at baseline—and with increasing CAG triplet repeat counts. The concentration at a given timepoint reflected the degree of motor and cognitive impairment as well as global and regional brain volumes. NfL in plasma was a prognostic indicator for disease onset within 3 years in participants who were premanifest *HTT* mutation carriers at baseline, independently of previously known prognostic factors. Furthermore, it was indicative of the likely rate of worsening of cognition, functional ability, and brain atrophy beyond age and CAG repeat count. We are unaware of other substances in blood, or for that matter in CSF, that have shown similarly strong prognostic power longitudinally and across a broad range of clinical, functional, and imaging measures. Our findings suggest that NfL concentrations in plasma offer a rapid and accessible means of assessing and predicting neuronal damage in people with Huntington's disease.

The closer association between NfL concentrations in plasma and the rate of whole-brain atrophy than with the striatal atrophy rate suggests that this factor better reflects the global rate of neuronal damage. Whole-brain change is well described in all stages of Huntington's disease, although the striatum undergoes proportionately greater atrophy in the early stages.^5^ A blood marker of neuronal damage across the whole brain that would be expected to respond to amelioration of CNS pathology would be extremely helpful for therapeutic development. The striking association we found between NfL concentrations in plasma and *HTT* CAG repeat count establishes a genetic dose–response connection in Huntington's disease.

This study allowed assessment of the longitudinal prognostic power of NfL concentrations in plasma. We were able to analyse in detail the associations with NfL from before disease onset and during manifest disease supported by rigorous clinical, cognitive, and neuroimaging data. Additionally, we were able to replicate the principal plasma findings in an independent cohort and confirm that concentrations in CSF are increased in individuals with *HTT* mutations, as has been previously reported.^8^, ^9^, ^15^, ^16^, ^17^ We further showed that NfL concentrations in plasma and CSF are closely correlated, which affirms the likely CNS origin of the NfL detected in both biofluids and suggests that NfL in blood could be a reliable estimator of concentrations in CSF.

Our study is not without limitations. First, some of the cross-sectional and longitudinal correlations of NfL with existing outcome measures are slight, probably due to both biological and measurement variability. Accurate quantification of putaminal atrophy, for example, is particularly challenging. One potential advantage of measuring NfL is that repeated assessment is not needed to indicate the rate of change in the brain at a given timepoint. Thus, modest associations in a natural history study do not preclude interpretable changes in NfL concentrations in plasma in response to an intervention that ameliorates neuronal damage. Second, the analysis of the independent CSF cohort was not powered to compare the relative effect sizes of NfL concentrations in CSF and plasma and, therefore, we could not determine whether measurement in plasma is a sufficient alternative or whether there remains an additional value in quantification in CSF. Third, we do not yet have longitudinal data on NfL concentrations in CSF or predictive power of this measurement for Huntington's disease progression. Fourth, TRACK-HD did not include participants with advanced Huntington's disease, and further study is needed to understand the patterns of NfL concentrations across the whole disease spectrum. To address these issues and to enable head-to-head comparison of NfL with other proposed biochemical markers, we have recruited 80 participants in whom NfL concentrations in CSF will be measured longitudinally, supported by neuroimaging,^28^ and have launched a multisite CSF study, HDClarity (NCT02855476), that will include 600 participants with premanifest to advanced Huntington's disease and controls. Finally, we note that although NfL was a strong predictor of onset and progression overall in this study, its variability was too great to allow confident prediction in individuals. Moreover, the clinical relevance of any predicted changes cannot be inferred from this work.

Measurement of NfL concentrations in plasma yielded promising results as a prognostic blood biomarker of onset, progression, and neuronal damage in Huntington's disease. We suggest that this approach has a potential role, once validated to regulatory standards, in facilitating development of novel disease-modifying therapeutics and, possibly, guiding treatment decisions once such treatments become available. We recommend that quantification of NfL concentrations in plasma be included in future observational and therapeutic trials for Huntington's disease. Retrospective analysis in blood samples collected in previous trials might also be useful, to test for evidence that interventions had effects on neuronal damage, even if the clinical outcomes were negative.
