# Corrections

**DOI:** 10.1016/S1474-4422(17)30255-7

**Published:** 2017-09

*Byrne LM, Rodrigues FB, Blennow K, et al. Neurofilament light protein in blood as a potential biomarker of neurodegeneration in Huntington's disease: a retrospective cohort analysis.* Lancet Neurol *2017; **16**: 601–09*—In figure 5C, the legend for the x-axis should have been “NfL concentration in plasma (pg/mL)” These corrections have been made to the online version as of July 14, 2017.

