# Supplementary material for: Neurofilament light protein in blood as a potential biomarker of neurodegeneration in Huntington's disease: a retrospective cohort analysis
Source: Lancet Neurol. 2017 Aug;16(8):601–9. doi: 10.1016/S1474-4422(17)30124-2 (PMC5507767; doi:10.1016/S1474-4422(17)30124-2)
Supplement: Supplementary appendix [file mmc1.pdf]

THE LANCET
Neurology

Supplementary appendix

This appendix formed part of the original submission and has been peer reviewed. We post it as supplied by the authors.

Supplement to: Byrne LM, Rodrigues FB, Blennow K, et al. Neurofilament light protein in blood as a potential biomarker of neurodegeneration in Huntington's disease: a retrospective cohort analysis. *Lancet Neurol* 2017; published online June 7. http://dx.doi.org/10.1016/S1474-4422(17)30124-2.

Neurofilament light protein in blood as a potential biomarker of neurodegeneration in Huntington's disease: a retrospective cohort analysis

Supplementary Appendix

Lauren M Byrne MRes, Filipe B Rodrigues MD, Prof Kaj Blennow PhD, Prof Alexandra Durr PhD, Prof Blair R Leavitt PhD, Prof Raymund A C Roos PhD, Rachael I Scahill PhD, Prof Sarah J Tabrizi PhD, Prof Henrik Zetterberg PhD, Prof Douglas Langbehn PhD, and Edward J Wild PhD

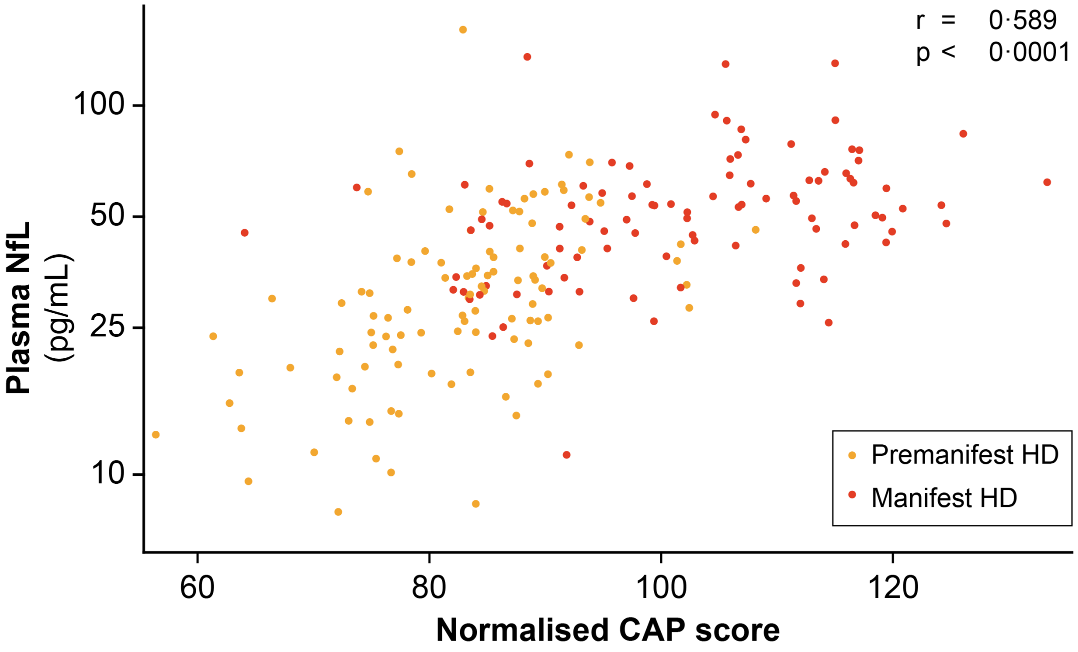

Supplementary Figure 1. Relationship between plasma NfL and normalised CAG-age product (CAP) score in HD mutation carriers. The normalised CAP score (Ross *et al.*, Nat Rev Neurol 2014; 10(4): 204-16) is an estimate of an individual's lifetime exposure to mutant huntington based on age and CAG repeat length. Given by **CAP = 100 × age × (CAG - 30) ÷ 627**, it is closely related to the 'disease burden score' of Penney *et al*. (Ann Neurol 1997; 41(5):689-92) but standardises such that a score of 100 represents the predicted age of onset from the conditional probability model of Langbehn *et al.* (Clin Genet 2004; 65(4):267-77).

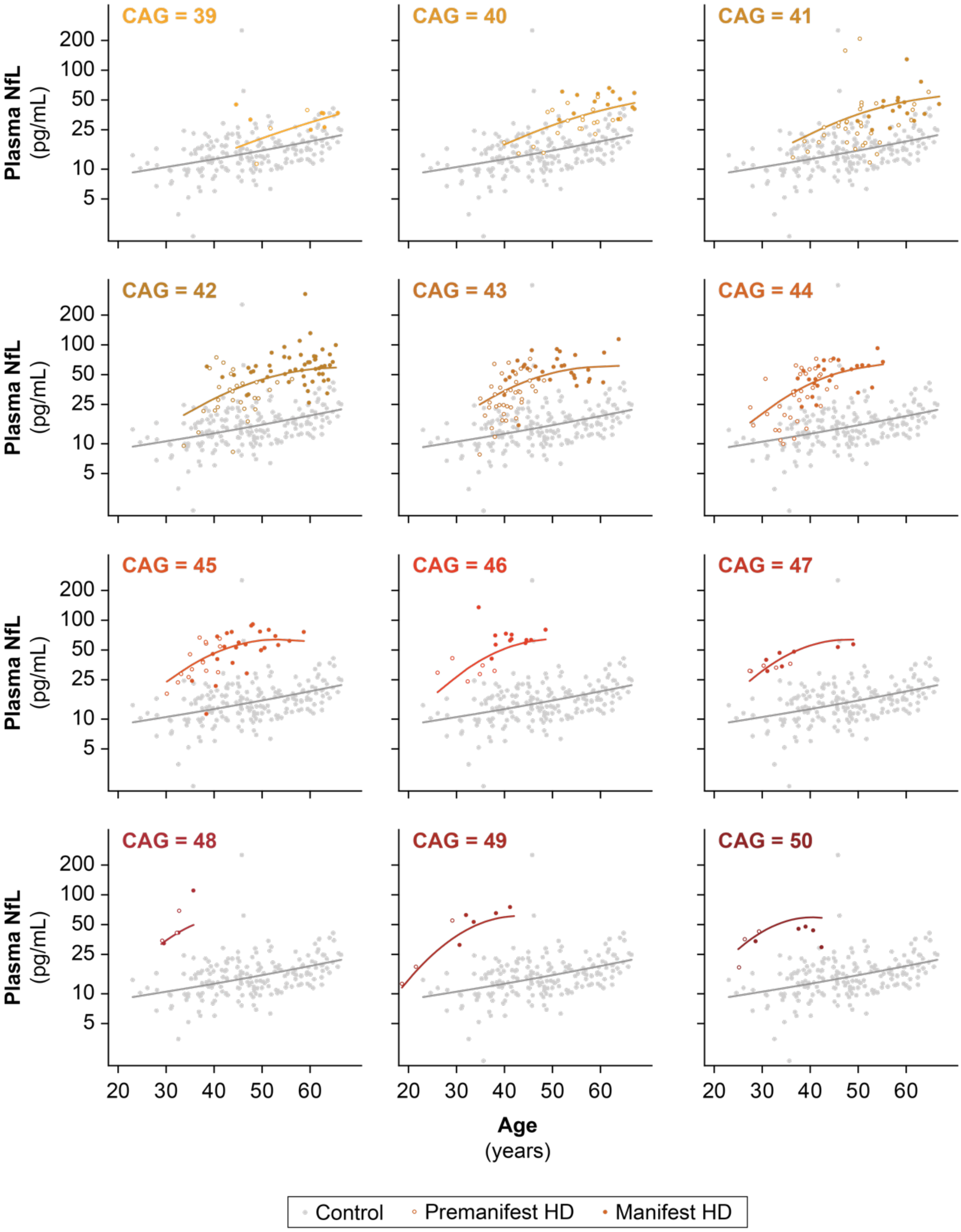

Supplementary Figure 2. Relationship between plasma NfL and age for each individual CAG repeat count and controls, modelled as per Figure 1B.

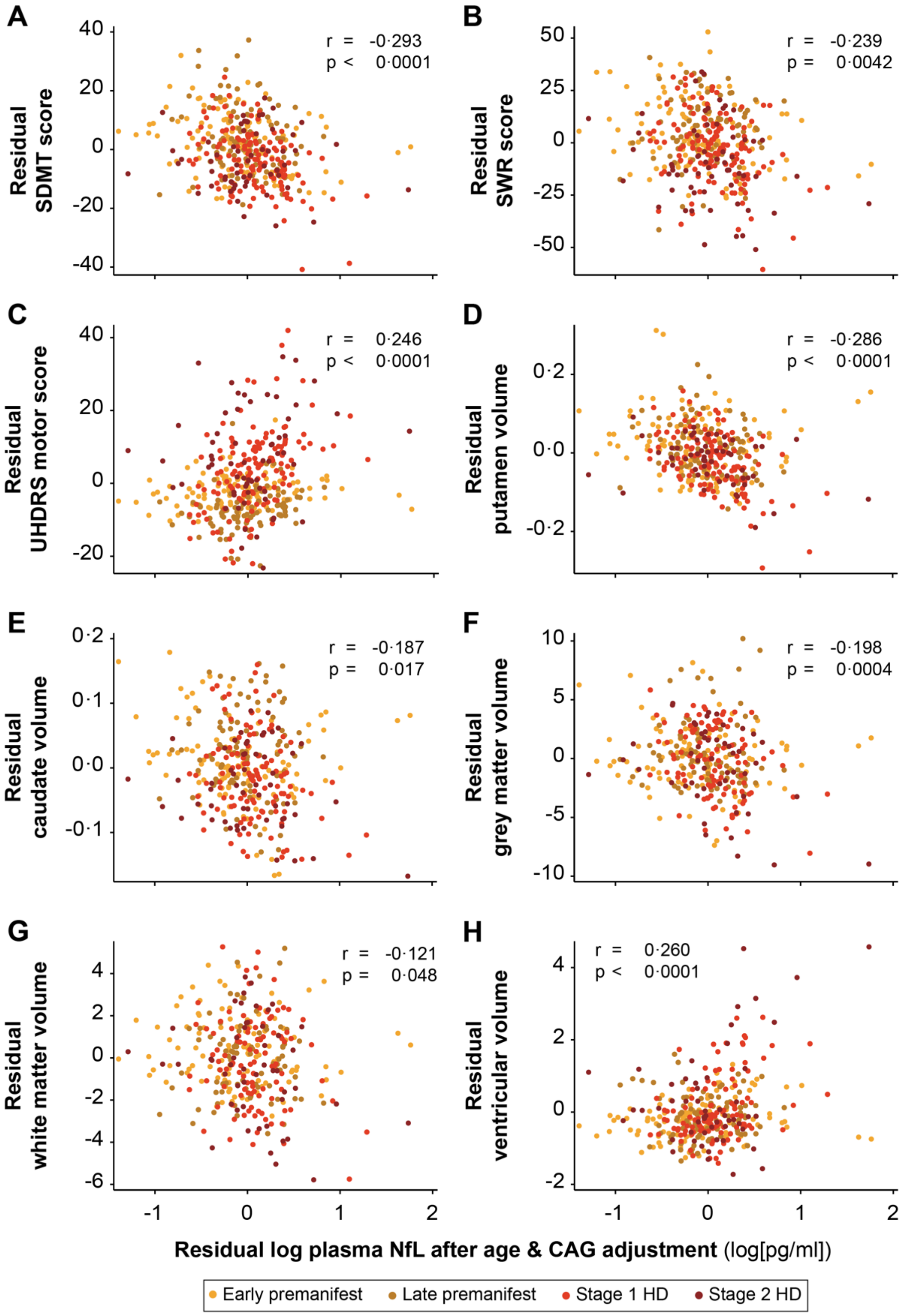

Supplementary Figure 3. Relationship between cross-sectional clinical and imaging measures after adjustment for age and CAG repeat length. Both axes show the residuals from the generalized linear model incorporating significant terms from the age-CAG analysis described in **Supplementary Table 3**. Abbreviations and vertical axis units are as per Figure 2.

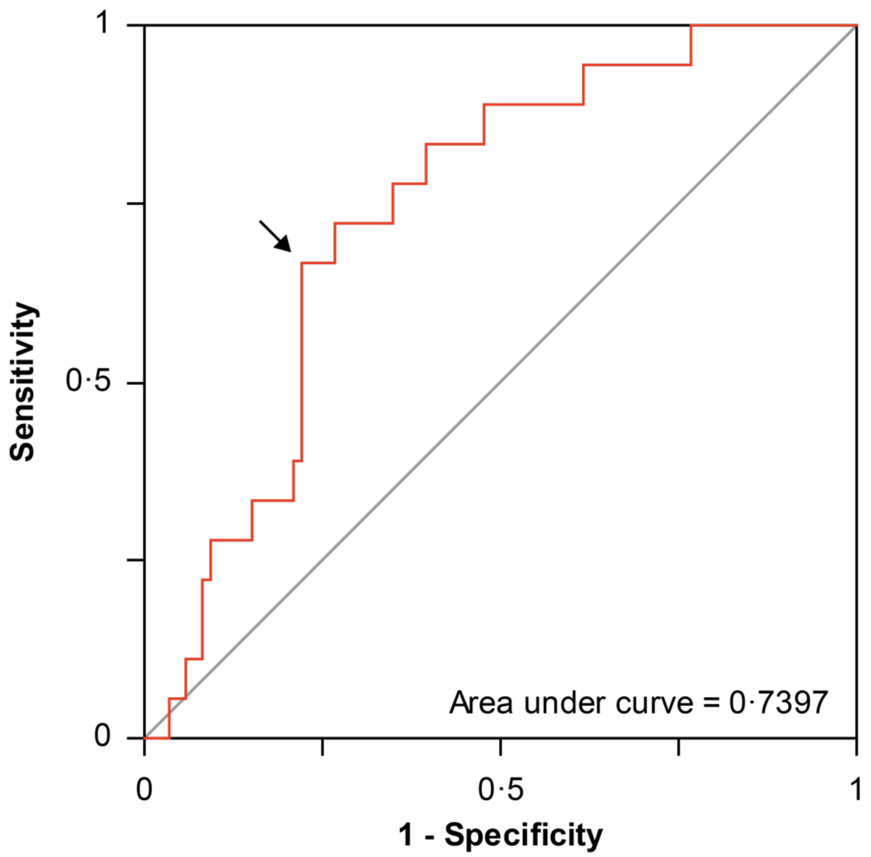

Supplementary Figure 4. Receiver operating characteristic (ROC) curve showing diagnosis risk within 36 months in the premanifest cohort. The point of highest mean sensitivity (0·667) and specificity (0·779), indicated by arrow, was at plasma NfL=3·61 log[pg/ml], close to the premanifest HD median value of 3·69.

Supplementary Table 1. Baseline characteristics of each cohort. A. TRACK-HD cohort. B. CSF cohort. Values are mean ± SD. CAG, CAG triplet repeat count; TMS, total motor score; TFC, total functional capacity; CAP, standardised CAG-age product score; 5yr onset prob, conditional probability of onset within 5 years (Langbehn et al, Am J Hum Genet 2009: 135B, 397-408). Disease duration is based on a rater's estimation of disease onset. Disease duration was available for 90 of the TRACK-HD manifest HD subjects. *pooled HD group includes 3 premanifest mutation carriers.

**A. TRACK-HD cohort**						**B. CSF cohort**			
**Group**	**Control**	**Early premanifest**	**Late premanifest**	**HD Stage 1**	**HD Stage 2**	**Control**		**HD***	
**n**	97	58	46	66	31	14	(13 plasma)	23	(17 plasma)
**Age**	46·08 ± 9·91	41·22 ± 8·52	40·68 ± 8·94	47·70 ± 10·32	51·29 ± 8·23	43·52 ± 13·17		50·11 ± 11·28	
**Sex (M:F)**	41:56	26:32	22:24	27:39	17:14	04:10		10:13	
**CAG**	N/A	42·14 ± 1·84	44·17 ± 2·39	43·62 ± 3·37	43·55 ± 2·46	N/A		43·6 ± 2·43	
**TMS**	1·51 ± 1·63	2·19 ± 1·42	2·96 ± 1·89	19·33 ± 9·28	30·29 ± 9·82	N/A		28·65 ± 22·11	
**TFC**	12·99 ± 0·10	12·90 ± 0·41	12·76 ± 0·78	12·27 ± 0·85	8·74 ± 1·06	13 ± 0		10·04 ± 3·04	
**CAP**	N/A	77·62 ± 7·88	88·93 ± 6·75	99·26 ± 12·67	108·54 ± 12·41	N/A		106·15 ± 19·20	
**5yr onset prob**	N/A	0·14 ± 0·061	0·32 ± 0·085	0·44 ± 0·16	0·53 ± 0·15	N/A		N/A	
**Disease duration**	N/A	N/A	N/A	5·53 ± 6·68	8·74 ± 4·68	N/A		N/A	

Supplementary Table 2. Baseline measures in the Track-HD cohort. A. NfL, clinical and imaging measures. **B.** Relationships between plasma NfL and CAP score and 5-year conditional onset probability. **C.** Inter-group differences, confidence intervals and effect sizes for baseline plasma NfL. Effect size is least square mean difference scaled by residual standard deviation in the underlying ANOVA model. CAP score, normalised CAG-age product score.

A		Control	Early premanifest	Late premanifest	HD stage 1	HD stage 2
	**n**	97	58	46	66	31
	**Plasma NfL**	18·11 ± 25·61	28·36 ± 22·24	39·39 ± 14·19	52·18 ± 20·52	57·48 ± 23·82
	**log plasma NfL**	2·68 ± 0·52	3·17 ± 0·56	3·61 ± 0·37	3·89 ± 0·35	3·96 ± 0·48
	**SDMT**	53·54 ± 8·99	52·83 ± 9·69	49·8 ± 11·36	37·35 ± 9·14	31·00 ± 9·42
	**SWR**	107·07 ± 16·29	102·19 ± 15·43	96·70 ± 17·55	84·56 ± 15·01	70·19 ± 19·87
	**Whole-brain**	81·30 ± 3·61	80·57 ± 3·63	78·54 ± 4·41	76·19 ± 4·57	71·69 ± 3·94
	**Caudate**	0·55 ± 0·058	0·48 ± 0·066	0·45 ± 0·073	0·38 ± 0·076	0·33 ± 0·06
	**Putamen**	0·70 ± 0·075	0·62 ± 0·10	7·42 ± 1·15	0·46 ± 0·93	0·43 ± 0·055
	**Grey matter**	46·36 ± 3·61	46·1 ± 2·92	669·29 ± 77·01	42·98 ± 3·39	40·69 ± 3·74
	**White matter**	32·94 ± 1·88	32·47 ± 1·53	31·08 ± 1·83	30·14 ± 2·14	28·78 ± 2·22
	**Ventricles**	1·12 ± 0·65	1·11 ± 0·53	1·26 ± 0·59	1·77 ± 0·84	2·36 ± 1·25

B		n	Pearson r	p-value
	**CAP score**	201	0·589	<0·0001
	**5yr onset prob**	201	0·589	<0·0001

C	Group 1	Group 2	Difference Between Means	95% Confidence Limits		Effect Size	P-value
	**Control**	**Early premanifest**	-0·49	-0·644	-0·336	2·21	<0·0001
	**Control**	**Late premanifest**	-0·928	-1·094	-0·762	4·18	<0·0001
	**Control**	**HD stage1**	-1·213	-1·36	-1·065	5·46	<0·0001
	**Control**	**HD stage2**	-1·276	-1·467	-1·084	5·75	<0·0001
	**Early premanifest**	**Late premanifest**	-0·438	-0·621	-0·255	1·97	<0·0001
	**Early premanifest**	**HD stage1**	-0·722	-0·889	-0·556	3·25	<0·0001
	**Early premanifest**	**HD stage2**	-0·785	-0·992	-0·579	3·54	<0·0001
	**Late premanifest**	**HD stage1**	-0·285	-0·463	-0·106	1·28	<0·0001
	**Late premanifest**	**HD stage2**	-0·348	-0·563	-0·132	1·57	0·0007
	**HD stage1**	**HD stage2**	-0·063	-0·265	0·139	0·28	0·5115

Supplementary Table 3 Relationships between plasma NfL, age and CAG repeat. A. Relationship between plasma NfL (log), age and *HTT* CAG repeat count in HD mutation carriers, examined using a polynomial function allowing for interactions with these predictors, their squares and all potential interactions. Age was centred at 50 years; CAG count at 42. DF, 198 degrees of freedom. **B.** Relationship between plasma NfL (log) and age in controls, 96 residual degrees of freedom. The relationship is essentially linear. (The nonsignificant Age^2 term is retained for consistency with the above model for participants with *HTT* CAG expansion.)

A

Effect	Estimate	Standard Error	t Value	P value
**Intercept**	3·8007	0·03814	99·65	<.0001
**Age**	0·03408	0·003735	9·12	<.0001
**CAG**	0·1866	0·02227	8·38	<.0001
**Age × CAG**	-0·00456	0·002469	-1·85	0·0661
**Age^2**	-0·00106	0·00036	-2·95	0·0036
**CAG^2**	-0·02331	0·006174	-3·78	0·0002
**Age^2 × CAG**	-0·00026	0·000105	-2·47	0·0143
**Age × CAG^2**	-0·00091	0·000279	-3·24	0·0014

B

Effect	Estimate	Standard Error	t Value	P value
**Intercept**	2·7463	0·05044	54·45	<.0001
**Age**	0·02061	0·004162	4·95	<.0001
**Age^2**	0·000066	0·000333	0·2	0·8435

Supplementary Table 4. A. Further detail of survival analysis in premanifest cohort. **B. Tests of equality in the survival analysis using log-rank and Wilcoxon tests.** P values given are asymptotic approximations. An exact permutation method for real-valued log-rank scores (exactRankTests v0.8-29 package for R v3.3.3), gave a 2-sided p value of 0.00247. **C. Log hazard ratio for the prediction of new diagnosis as a function of log plasma NfL concentrations after controlling for other known predictors.** Analysis was done in the group that was premanifest at baseline. Due to the limited number of new diagnoses (18 over 36 months), covariate adjustments were controlled one at a time, except for age, CAG length, and their interaction. The log NfL hazard ratio is little changed by additional of any of these covariates, suggesting that NfL is a risk predictor that is not redundant with other known predictors. [a]For context, the standard deviation of log NfL concentration in at-risk preHD subjects was 0·39 log(pg/ml) (See **Supplementary Table 2**).

A	Time (yrs)	Survival	Failure	Survival SE	Failed	Remaining	95% CI	
	**NfL above median**							
	0	1.000	0.000	0	0	52	_	_
	1	0.923	0.077	0.037	4	48	0.851	0.996
	2	0.827	0.173	0.0525	9	43	0.724	0.930
	3	0.712	0.289	0.0628	15	37	0.588	0.835
	**NfL below median**							
	**Time (yrs)**	**Survival**	**Failure**	**Survival SE**	**Failed**	**Remaining**	**95% CI**	
	0	1.000	0.000	0	0	52	_	_
	1	0.981	0.019	0.019	1	51	0.944	0.999
	3	0.942	0.058	0.0323	3	49	0.879	0.999

B	Test	Chi-Square	P value
	**Log-Rank**	9.720	0.0018
	**Wilcoxon**	9.721	0.0018

C	Covariate controlled	NfL log Hazard Ratio per log(pg/ml)[a]	Standard error	p-value
	**None**	1·192	0·409	0·0036
	**Age, CAG, Age × CAG**	1·109	0·532	0·0371
	**Caudate volume**	1·084	0·451	0·0162
	**Putamen volume**	1·213	0·548	0·0269
	**Whole-brain volume**	1·060	0·436	0·0151
	**Ventricular volume**	1·119	0·435	0·0101
	**White-matter volume**	1·052	0·453	0·0201
	**Grey-matter volume**	1·092	0·455	0·0163
	**UHDRS Total Motor Score**	0·866	0·451	0·0549
	**Speeded tapping mean inter-tap time (nondominant hand)**	1·318	0·406	0·0011
	**Symbol-digit modality test**	0·923	0·449	0·0398
	**Indirect circle tracing time**	1·512	0·471	0·0013
	**Paced tapping at 3 Hz (inverse standard deviation)**	0·990	0·472	0·0358
	**Spot-the-Change**	1·152	0·445	0·0096

Supplementary Table 5. The prognostic value of baseline NfL for longitudinal brain volume change in premanifest and manifest HD. After examining longitudinal predictive power of baseline plasma NfL for longitudinal brain volume change measures across all mutation carriers, it was re-examined controlling for group (premanifest or manifest HD) to examine whether plasma NfL offers additional prognostic power beyond that of group status. Where these analyses were significant, we then examined for the interaction between premanifest and manifest HD to determine whether there was a significantly different relationship with NfL between the two groups. Such relationships were identified for whole-brain and grey matter atrophy and lateral ventrical expansion, all of which were more strongly predicted by NfL in the manifest HD group. Regression estimate represents the least square mean slope for preHD and early HD. Unit for 'estimate' is percent TIV change per log(pg/ml) plasma NfL. * represents the group with the signifcantly stronger NfL association. For context, the standard deviation of log NfL concentration in preHD subjects was 0·39 log(pg/ml) and in early HD was 0·52log(pg/ml) (see **Supplementary Table 2**).

**Longitudinal measure**	**Adjusting for age, CAG and group**		**PreHD vs Early HD Interaction**					
	**Regression estimate**	**p-value**	**PreHD slope estimate**	**p-value**	**Early HD slope estimate**	**p-value**	**Estimate difference**	**p-value**
Putamen	0·00039	0·8161	0·00016	0·928	0·00061	0·816	0·00045	0·884
Caudate	0·00134	0·0279	0·00177	0·008	0·00091	0·343	0·00086	0·436
Whole brain	0·205	<0·0001	0·129	0·0007	0·282*	<0·0001	0·153	0·043
White matter	0·08	<0·0001	0·048	0·004	0·112	0·002	0·064	0·082
Grey Matter	0·077	<0·0001	0·011	0·496	0·144*	<0·0001	0·143	< 0·0001
Lateral Ventricle	-0·0606	<0·0001	-0·024	0·004	-0·097*	<0·0001	0·073	0·002

The TRACK-HD Investigators

Australia: C Campbell, M Campbell, E Frajman, I Labuschagne, C Milchman, A O'Regan, J Stout (Monash University, VIC). Canada: A Coleman, R Dar Santos, J Decolongon, A Sturrock (University of British Columbia, Vancouver, BC). France: E Bardinet, C Jauffret, D Justo, S Lehericy, C Marelli, K Nigaud, R Valabrègue (APHP, Hôpital Salpêtriere, Paris). Germany: N Bechtel, S Bohlen, R Reilmann (University of Münster, Münster); B Landwehrmeyer (University of Ulm, Ulm); A Hoffman, P Kraus (University of Bochum, Bochum). Netherlands: SJA van den Bogaard, E M Dumas, J van der Grond, EP t'Hart, C Jurgens, M-N Witjes-Ane (Leiden University Medical Centre, Leiden). UK: N Arran, J Callaghan, D Craufurd, C Stopford (St Mary's Hospital, Manchester); C Frost, R Jones (London School of Hygiene & Tropical Medicine, London); H Crawford, N C Fox, C Gibbard, N Hobbs, N Lahiri, I Malone, R Ordidge, G Owen, T Pepple, J Read, M J Say, D Whitehead (University College London, London); S Keenan (Imperial College London, London); D M Cash (IXICO, London); C Berna, S Hicks, C Kennard (University of Oxford, Oxford). USA: T Acharya, E Axelson, H Johnson, D Langbehn, C Wang, (University of Iowa, Iowa City, IA); B Borowsky (CHDI Foundation, New York, NY); S Lee, W Monaco (Massachusetts General Hospital, Boston, MA); C Campbell, S Queller, K Whitlock (Indiana University, Bloomington, IN).
